# Less efficient detection of positive facial expressions in parents at risk of engaging in child physical abuse

**DOI:** 10.1186/s40359-019-0333-9

**Published:** 2019-08-27

**Authors:** Koji Shimada, Ryoko Kasaba, Akiko Yao, Akemi Tomoda

**Affiliations:** 1 Research Center for Child Mental Development, University of Fukui, 23-3 Matsuoka-Shimoaizuki, Eiheiji-cho, Yoshida-gun, Fukui, 910-1193 Japan; 2 Biomedical Imaging Research Center, University of Fukui, 23-3 Matsuoka-Shimoaizuki, Eiheiji-cho, Yoshida-gun, Fukui, 910-1193 Japan; 3 Division of Developmental Higher Brain Functions, United Graduate School of Child Development, University of Fukui, 23-3 Matsuoka-Shimoaizuki, Eiheiji-cho, Yoshida-gun, Fukui, 910-1193 Japan; 4 Department of Child and Adolescent Psychological Medicine, University of Fukui Hospital, 23-3 Matsuoka-Shimoaizuki, Eiheiji-cho, Yoshida-gun, Fukui, 910-1193 Japan

**Keywords:** Child physical abuse, Physical punishment, Social information processing, Happy face detection, Face-in-the-crowd task

## Abstract

**Background:**

Parental physical punishment (e.g., spanking) of children can gradually escalate into child physical abuse (CPA). According to social-information processing (SIP) models of aggressive behaviors, distorted social cognitive mechanisms can increase the risk of maladaptive parenting behaviors by changing how parents detect, recognize, and act on information from their social environments. In this study, we aimed to identify differences between mothers with a low and high risk of CPA regarding how quickly they detect positive facial expressions.

**Methods:**

Based on their use of spanking to discipline children, 52 mothers were assigned to a low- (*n* = 39) or high-CPA-risk group (*n* = 13). A single-target facial emotional search (face-in-the-crowd) task was used, which required participants to search through an array of faces to determine whether a target emotional face was present in a crowd of non-target neutral faces. Search efficiency index was computed by subtracting the search time for target-present trials from that for target-absent trials.

**Results:**

The high-CPA-risk group searched significantly less efficiently for the happy, but not sad, faces, than did the low-CPA-risk group; meanwhile, self-reported emotional ratings (i.e., valence and arousal) of the faces did not differ between the groups.

**Conclusions:**

Consistent with the SIP models, our findings suggest that low- and high-CPA-risk mothers differ in how they rapidly detect positive facial expressions, but not in how they explicitly evaluate them. On a CPA-risk continuum, less efficient detection of positive facial expressions in the initial processes of the SIP system may begin to occur in the physical-discipline stage, and decrease the likelihood of positive interpersonal experiences, consequently leading to an increased risk of CPA.

## Background

A general definition of the physical punishment of children, such as spanking (i.e., open-handed swats to the buttocks or extremities), is “the use of physical force with the intention of causing a child to experience pain, but not injury, for the purpose of correction or control of the child’s behavior” [1]. However, for children, receiving physical punishment has been associated with cognitive-behavioral, physical, and mental health problems in later life [2–6]; further, it has also been suggested to alter the trajectories of brain development [7, 8]. Given such long-term adverse consequences, physical punishment (e.g., spanking) can be defined as a form of child maltreatment, which encompasses a spectrum of abusive actions (physical, emotional, sexual abuse) or lack of actions (i.e., neglect) by the parent or other caregivers. Indeed, spanking has empirically loaded on the same factor structure with physical and emotional abuse items which indicates a similar underlying construct to physical and emotional abuse [9].

In recent years, the traditional perceived dichotomy between physical punishment and child physical abuse (CPA) has begun to disappear [10], and physical punishment is beginning to be considered a risk factor of CPA. Specifically, it is believed to escalate gradually into CPA, following a continuum ranging from positive (effective) discipline, to physical punishment, to abusive treatment [11–14]. Today, physical punishment in all settings, including the home, is legally prohibited in 56 countries around the world [15]. However, to prevent child maltreatment and related problems (e.g., co-parental conflicts), it is of particular importance to better understand the social cognitive mechanisms that prompt a parent to progress from positive discipline along the continuum towards physical punishment and/or CPA.

According to social-information processing (SIP) models regarding CPA risk [16–21], distorted social cognitive mechanisms may increase the risk of maladaptive parenting behaviors by changing how parents detect, recognize, and act on information from social environments. In Milner’s [19, 20] studies, social cognitive mechanisms are assumed to encompass four stages: first, perceiving social behavior (e.g., facial expressions); second, interpreting and evaluating the meanings of the behavior; third, integrating the information and selecting a response; and fourth, implementing and monitoring the response. These cognitive processing stages are also assumed to be influenced by cognitive schemata that are developed through experience and stored in long-term memory. When encountering a discipline situation, a parent at risk of engaging in CPA is likely to inaccurately perceive the child’s behavior, consider the behavior to be hostile (aggressive) and construct a negative narrative regarding the causes of the behavior. For example, high-CPA-risk parents tend to view negative child behaviors as being due to internal, stable, and global child factors and being motivated by hostile (aggressive) intent [20]. Various sources [22] show that parents with a higher CPA risk are more likely to show greater processing of negative (i.e., angry, hostile) stimuli in the SIP system in regard to schema accessibility [23–25], attentional control [26], interpretation [27–29], attribution [30, 31], and subjective feelings [32], although a few studies [33] have found that less, rather than greater, accessibility to negative information is present in parents with a higher CPA risk. Overall, the main findings of prior research have suggested that greater processing of negative stimuli in the SIP system increase the likelihood of parents engaging in aggressive behaviors [22].

In addition to altered negative processing, the parents with a high CPA risk, relative to low-risk parents, also seem to exhibit less processing of positive (i.e., happy, benign) stimuli in the SIP system [23, 24, 27, 33]. Aggression may be associated with the twice the challenges, including both the altered processing of angry (hostile) stimuli and happy stimuli in the SIP system. However, relatively less attention has focused on the decreased processing of positive information, including schema accessibility [23, 24, 33] and interpretation [27]. For example, Crouch et al. [24] reported that, in a cued-recall task, high-CPA-risk parents, compared to low-CPA-risk parents, recalled less child-care information when cued by positive terms, indicating less accessibility of positive schema stored in long-term memory. Similarly, Dopke et al. [27] found that low-CPA-risk parents, unlike high-CPA-risk parents, have positive interpretive tendencies regarding child behaviors. As positive social information (e.g., a happy facial expression) has important adaptive functions, such as by facilitating interpersonal relationships [34, 35], efficiently perceiving and interpreting such critical can secure important interpersonal benefits (e.g., parent-child attachment formation). In a parent-child communicative setting, detection of a child’s happiness engenders happiness in the perceiving parent, facilitating a feedback loop, whereby the detecting of happiness leads to the parent having a happy experience, and the parent’s consequent expression of happiness elicits further happiness in the child.

In the current study, we mainly focused on detection efficiency (i.e., initial processes of the SIP system) of positive information in the low- and high-CPA risk parents, rather than the schema accessibility and interpretation focus of previous studies [23, 24, 27, 33]. In particular, we examined differences between parents (mothers) with low and high risks of engaging in CPA in relation to their speed of detection of positive (happy) facial expressions. We hypothesized that high-CPA-risk mothers would exhibit lower performance on the happy face detection task than on the low-CPA-risk mothers. To determine the CPA risk, we focused on the use of spanking (i.e., swatting a child’s buttocks or extremities with an open hand) as a form of discipline. Consequently, mothers who never spanked their children in order to discipline them were classified as low CPA risk, and mothers who spanked their children to discipline them were classified as high CPA risk, which was based on the Index of Child Care Environment (ICCE) [36] that was developed using the Home Observation for Measurement of Environment (HOME) [37]. As an experimental detection paradigm, a single-target face-search task (i.e., a face-in-the-crowd task) was used, which required participants to search through an array of schematic faces to determine whether a target happy face was present in a crowd of non-target neutral faces [38, 39]. As target-absent trials require an exhaustive search of the entire array before participants can indicate that the target is absent, the task responses provide an important baseline for the responses in target-present trials [38, 40]. The response differences between the target-present and target-absent trials indicate the level of efficiency regarding searching for happy faces, with higher values indicating greater search efficiency. In the single-target face-search tasks, we used not only the happy-face search task but also the sad-face search task, which allowed us to take into consideration visual (physical) saliency for the target face among the non-target faces. From an evolutionary perspective, mothers who could efficiently detect child’s sad expressions as signs of distress might provide a better chance of survival for the premature child [41–43]. In particular, greater processing of a child’s sad expressions has been shown in neglectful than non-neglectful parents [44], but not shown in physically abusive (high-CPA-risk) parents [45], suggesting differences in distorted social cognitive mechanisms underlying physically abusive and neglectful parenting behaviors. Based on previous studies [22, 44, 45], we hypothesized that higher CPA risk would not be associated with the altered processing of sad stimuli in the SIP system. If our hypothesis was correct, high-CPA-risk mothers would exhibit lower search efficiency for the target-happy, but not for the target-sad faces than the low-CPA-risk mothers. Conversely, if a higher CPA risk was associated with the altered detection of visual saliency for the target face among the non-target faces, high-CPA-risk, relative to low-CPA-risk mothers, would exhibit lower search efficiency for the target-happy and target-sad faces, respectively.

## Methods

### Participants

Fifty-two healthy Japanese mothers (age range = 27–46 years; mean age = 35.5 years; SD = 4.2 years) who were caring for one or more young children participated in this study, after providing written informed consent. The study protocol was approved by the Ethics Committee of the University of Fukui and was conducted in accordance with the Declaration of Helsinki and the Ethical Guidelines for Clinical Studies published by the Ministry of Health, Labour, and Welfare of Japan. Almost all mothers (51 [98.1%]) were caring for at least one preschool child (one [1.9%] was caring for an elementary school child). All mothers had completed at least 12 years of education (non-compulsory secondary-level or post-school university-level education), which was categorized as a relatively high level of education [46]. Further, they were all living above the relative poverty line, which was set at 50% of the country’s median household income [47]. All had normal vision or corrected-to-normal vision. Moreover, through self-report questionnaires, they stated that they had no history of brain injury or neurological or psychiatric illness, and that were not currently using psychoactive medications.

Using the ICCE, the mothers were classified with respect to their CPA risk, based on their use of spanking to discipline children for misbehavior. In Japan, milder physical punishment such as spanking has been still considered a socially acceptable parental behavior [48]. For the ICCE subscale “avoidance of restriction” (two items: Q1 “what would you do if your child spilled milk on purpose?” and Q2 “how many times did you spank your child last week?”), the answers “I would not spank” and “I did not spank” were defined as low CPA risk, and the answers “I would spank” and/or “I did spank” were defined as high CPA risk. Of the 52 mothers, 39 (75%) were classified as low CPA risk, and the remaining 13 (25%) were classified as high CPA risk (approximately 8% of the High CPA risk group answered “I would spank” to Q1 and 92% answered “I did spank” to Q2).

### Measures of maternal characteristics

The Buss-Perry Aggression Questionnaire (BPAQ) [49, 50] was used to measure the mothers’ aggression; this consists of four subscales: anger, hostility, physical aggression, and verbal aggression. Meanwhile, to assess empathic ability, the Interpersonal Reactivity Index (IRI) [51, 52] was used, which is composed of four subscales (Empathic Concern, Personal Distress, Perspective-Taking, and Fantasy). Further, the Japanese version of the Parenting Stress Index (J-PSI) [53], which is an adaptation of the PSI [54], was used to evaluate the mothers’ parenting stress. The J-PSI is comprised of items on Child (reinforces parent, mood, acceptability, distractibility/hyperactivity, demandingness, problems/worries, and sensitivity to stimuli) and Parent domains (role restriction, social isolation, relationship with spouse, competence, depression, sad/uneasy feelings after leaving hospital, attachment, and health). The Beck Depression Inventory-II (BDI-II) [55, 56] was used to measure the mothers’ depressive symptoms, and the Parental Bonding Instrument (PBI) [57, 58] was used to obtain retrospective information on the parental caregiving behaviors the participants perceived during their first 16 years of life. The PBI is comprised of two fundamental dimensions of parental behaviors: parental emotional support (care) and parental protectiveness (protection).

### Stimuli

**Fig. 1:**
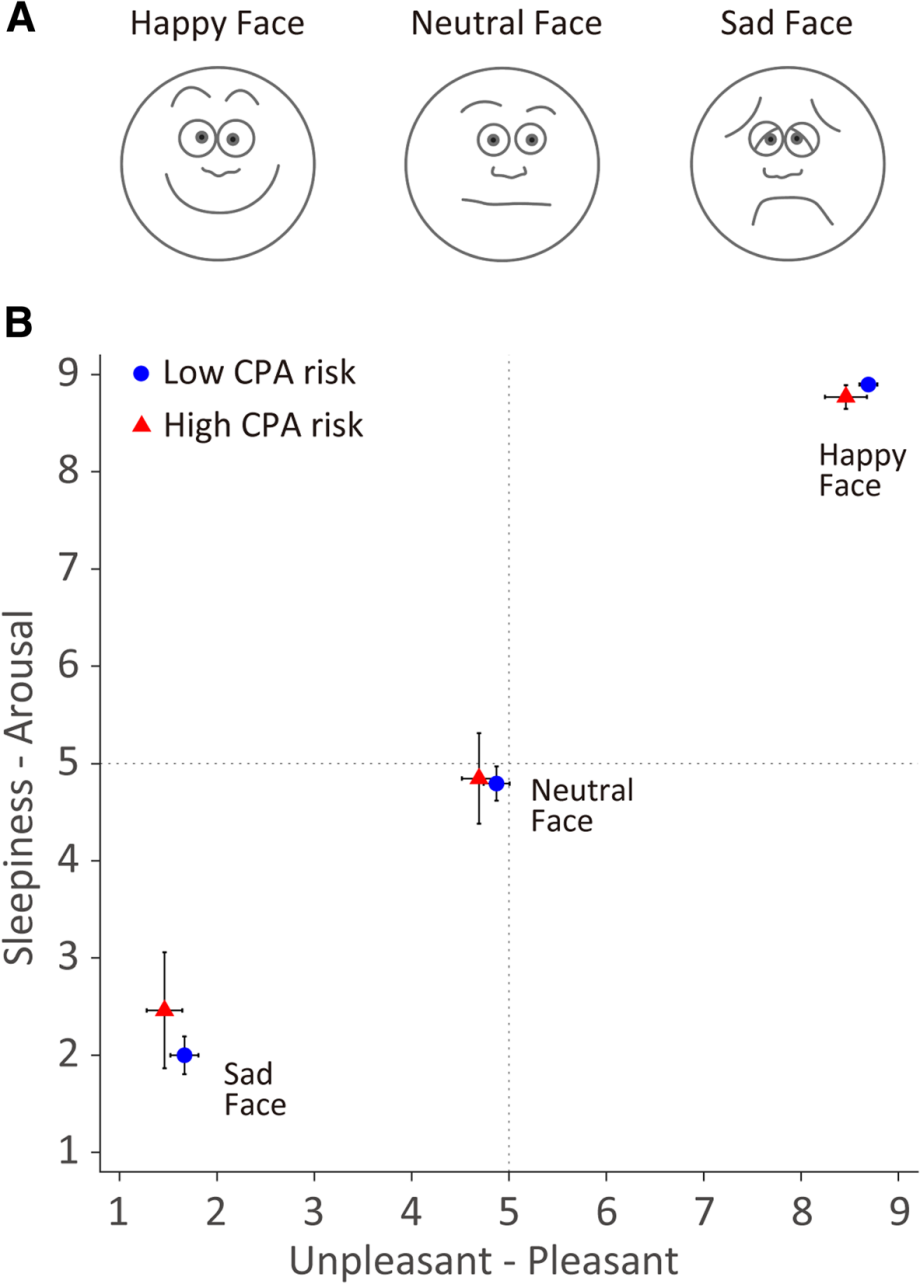
**a** Emotional schematic faces (i.e., happy, neutral, sad) selected from the Wong-Baker Faces [59]. **b** Mean valence and arousal ratings of the faces for the CPA-risk groups. Error bars represent the standard errors of the mean

The stimuli were three schematic images of facial emotions (happy, sad, neutral) (Fig. 1a) taken from the Wong-Baker Faces Pain Rating Scale (WBFS) [59]. The faces, including the outline, eyebrows, eyes, and mouth, were depicted using black lines on a white background. The happy face used in this study was taken from the WBFS smiling face representing “no hurt” (Face 0), while the sad face was taken from the WBFS sad face representing “hurts a whole lot” (Face 8). For the neutral face, Face 4 from the WBFS was used. Each face image was pasted onto a white background that was 175 × 165 pixels in size and assigned to any of 12 possible locations on a 4 × 3 array.

### Face ratings

Participants rated, using a nine-point Likert scale, each face image in terms of valence and level of arousal [60, 61]. For Valence ratings, the scale ranged from *extremely unpleasant* (1) to *extremely pleasant* (9), and for Arousal, the scale ranged from *extreme sleepiness* (1) to *extremely high arousal* [9]. On the arousal-valence orthogonal dimension of the circumplex model of affect [61], happiness is high in pleasantness and high in arousal, whereas sadness is low in pleasantness but low in arousal.

### Face-search task

The stimuli were displayed on a 14-in. monitor with a refresh rate of 60 Hz and a screen resolution of 1024 × 768 pixels and were presented using Presentation software (Neurobehavioral Systems, Albany, CA) running on a Windows computer. Participants were seated approximately 70 cm away from the monitor and gave responses using the left and right arrow keys on the computer’s keyboard. Before beginning the experiment, all participants received instructions and performed a short practice task.

Participants were instructed to perform, as quickly and accurately as possible, two visual search tasks (happy, sad); each task had three set-size conditions (1, 6, and 12); similar visual-search-task paradigms involving emotional schematic faces have been applied in several previous experimental psychological studies [38, 39]. In each task, our participants indicated whether a target face was present on the display by pressing the right or left direction arrow key. The right direction arrow key was associated with target-present detection, whereas the left direction arrow key was associated with target-absent (non-target) detection. In one of the visual search tasks, the target they searched for was always a happy face, and in the other, the target was always a sad face. For the first set size (set of 1), a target face or a distracting non-target neutral face was presented in only one of the 12 possible locations in a 4 × 3 array. For the second set size (set of 6), a target face and five non-target faces, or six non-target faces, were presented in six of the 12 possible locations. Finally, for the third set size (set of 12), a target face and 11 non-target faces, or 12 non-target faces, were presented in the 12 possible locations.

Participants completed six task blocks, each consisting of 24 trials, giving a total of 144 trials. Within each task block, half were target-present trials and half were target-absent trials. The task blocks were presented in order of ascending set size (1, 6, and 12). Each trial began with a black fixation cross presented in the middle of the screen, which remained on screen for 1000 ms. The face stimuli were then presented for 5000 ms or until the participant responded by pressing one of the two keys with the index or middle finger of the right hand. The next trial commenced after an inter-trial interval of 1000 ms.

### Visual saliency

A total of 144 visual scene images, including 36 happy-face-present, 36 sad-face-present, and 36 target-absent neutral (twice) scenes, were used for the face-search task experiment. For the three types of visual scenes (happy, sad, and neutral), visual saliency maps were computed according to the Graph-Based Visual Saliency (GBVS) model [62]. The GBVS algorithms extract low-level visual features (e.g., intensity, orientation), generate individual feature maps by extracting locations of distinctive features, and integrate these maps to generate an overall saliency map. The values of the saliency maps range from 0 to 1, depicting the distribution of visual saliency across the scene image. The saliency maps of the three types of visual scenes had comparable mean values (*F*(2, 105) = 0.13, *p* > .87), indicating control for the visual saliency among the three types of visual scenes (happy-face-present scenes, mean value [SD] = 0.152 [0.097]; sad-face-present scenes, mean value [SD] = 0.142 [0.095]; target-absent neutral scenes, mean value [SD] = 0.153 [0.097]).

### Data analysis

The mean response time (RT) and accuracy (percentage of correct responses) were calculated individually, using separate measures for the two trial types (target-present, target-absent), the two target emotions (happy, sad), and the three set sizes (1, 6, and 12). RTs were only analyzed for correct responses. Data for measures for which participants had an error rate in excess of 25% were excluded from each analysis. Search slope was calculated for each task by fitting a linear function to the mean RTs for the three set sizes. An increasing slope with more set sizes (distractors) indicated a serial exhaustive search strategy, whereas a flattened slope indicated a pre-attentive parallel search strategy. As target-absent trials require participants to perform an exhaustive search of the entire array before they can indicate that the target is absent, the RTs and slopes for these trials provided an important “baseline” against which the RTs and slopes for the target-present trials could be interpreted [38, 40]. Thus, differences in RT (Δ RT) and search slope (Δ search slope), which would reflect a search advantage (i.e., efficiency) regarding target-present over target-absent trial types, were calculated by subtracting the RTs and slopes of the target-present trial types from those of the target-absent (baseline) trial types. The RT differences (Δ RT) reflected the search efficiency at a specific set size, whereas the search slope differences (Δ search slope) reflected the overall search efficiency across the three set sizes. These differences in search efficiency create indexes with positive values when there is a search advantage (efficiency), and with negative values when there is a search disadvantage (inefficiency) regarding target-present relative to target-absent trial types. All statistical analyses were performed using SPSS Statistics (version 22; IBM Japan, Tokyo, Japan). The accuracy and Δ RT data were analyzed using a two-way analysis of variance (ANOVA) with one between-subjects factor (CPA risk: low, high) and one within-subject factor (set size: 1, 6, and 12). The Δ search slope data for the 2 CPA risk groups were analyzed using a two-tailed *t*-test. An alpha level of .05, with Bonferroni correction, when appropriate, was used for all significance tests.

## Results

### Demographic and psychological characteristic data

The demographic and psychological characteristics of the CPA groups are listed in Table 1. There were significant differences between the 2 CPA-risk groups for five measures: number of children, *t*(50) = 3.61, *p* < .001, *d* = 1.03; BPAQ Anger scores, *t*(50) = 2.48, *p* = .016, *d* = 0.75; J-PSI Child domain Mood subscore, *t*(50) = 2.78, *p* = .007, *d* = 0.92; J-PSI Child domain Acceptability subscore, *t*(50) = 2.68, *p* = .009, *d* = 0.78, and J-PSI Parent domain Attachment subscore, *t*(50) = 3.15, *p* = .002, *d* = 0.92. There were no differences between the remaining scores (all *p*s > .07).

**Table 1:** The Child Physical Abuse (CPA)-risk group characteristics

	Low CPA Risk (*n* = 39)			High CPA Risk (*n* = 13)		
	Mean	SD	%	Mean	SD	%
Demographic Characteristics						
Age (years)	35.6	(4.6)		35.2	(2.5)	
Education (≥ 12 years)			100.0			100.0
Married			97.4			100.0
Number of family members	4.4	(1.3)		5.2	(1.3)	
Number of children	1.9	(0.6)		2.6	(0.9)	
Time since last childbirth (months)	39.5	(21.3)		37.8	(18.5)	
Gender of child (female)			47.4			42.9
Health problems of child			30.8			46.2
Living above the relative poverty line			100.0			100.0
Buss-Perry Aggression Questionnaire						
Anger	13.9	(4.0)		17.2	(4.9)	
Hostility	15.3	(3.8)		17.0	(4.0)	
Physical aggression	12.6	(4.0)		14.9	(4.9)	
Verbal aggression	14.2	(2.9)		13.3	(2.4)	
Interpersonal Reactivity Index						
Perspective-taking	17.3	(3.4)		16.2	(3.7)	
Empathic concern	18.0	(3.2)		18.2	(2.7)	
Fantasy	13.6	(3.0)		13.3	(3.3)	
Personal distress	13.9	(4.4)		13.4	(5.0)	
Parenting Stress Index						
Child domain scores	85.1	(17.9)		97.5	(16.6)	
C1: Reinforces parent	11.1	(3.2)		12.9	(3.1)	
C2: Mood	18.5	(4.8)		22.5	(4.1)	
C3: Acceptability	10.0	(3.0)		12.9	(4.2)	
C4: Distractibility/Hyperactivity	14.8	(3.9)		16.3	(2.9)	
C5: Demandingness	12.9	(4.2)		12.8	(2.5)	
C6: Problems/worries	8.9	(3.1)		11.0	(4.6)	
C7: Sensitivity to stimuli	8.9	(3.4)		9.2	(2.0)	
Parent domain scores	103.1	(22.6)		112.1	(28.2)	
P1: Role restriction	20.3	(5.8)		21.6	(7.7)	
P2: Social isolation	16.0	(5.3)		17.3	(6.4)	
P3: Relationship with spouse	12.1	(5.4)		12.9	(5.7)	
P4: Competence	21.9	(3.7)		23.5	(3.6)	
P5: Depression	10.3	(3.6)		11.8	(3.9)	
P6: Sad/uneasy feeling after leaving hospital	8.7	(3.2)		7.9	(3.7)	
P7: Attachment	6.5	(2.2)		8.9	(3.1)	
P8: Health	7.5	(2.4)		8.2	(2.6)	
Beck Depression Inventory-II	11.2	(7.5)		14.7	(13.2)	
Parental Bonding Instrument						
Maternal care	25.1	(9.3)		24.4	(7.1)	
Maternal protection	12.3	(7.8)		10.4	(6.2)	
Paternal care	23.4	(8.9)		23.5	(6.3)	
Paternal protection	10.4	(7.2)		9.2	(5.8)	

### Face ratings data

As shown in Fig. 1b, the low- and high-CPA-risk groups gave similar Valence and Arousal ratings for all three face images (happy, sad, neutral) (all *ps* > .24). Overall, the happy face image was rated high in pleasantness and high in arousal, whereas the sad face image was low in pleasantness but low in arousal.

### Face-search-task data

#### Accuracy

Both the low- and high-CPA-risk groups showed over 90% accuracy for all trials (Table 2). For the happy-face search task, a two-way ANOVA was conducted on the target-present trial type with one between-subjects factor (CPA risk: low, high) and one within-subject factor (set size: 1, 6, and 12); it was determined that CPA risk had no effect on accuracy (*F*(1, 48) = 2.15, *p* > .14). There was a main effect of set size (*F*(2, 96) = 11.42, *p* < .001, η^*2*^_*p*_ = .192) and an interaction between the two factors (*F*(2, 96) = 4.66, *p* = .012, η^*2*^_*p*_ = .088). Subsequent comparisons for the simple main effect indicated that the high CPA risk participants showed less accuracy in the set of six than did those with low CPA risk (*t*(48) = 2.70, *p* = .009, *d* = 0.84). For the target-absent trial type, there were no effects for CPA risk (*F*(1, 48) = 1.37, *p* > .24), set size (*F* < 1), or an interaction effect (*F* < 1).

**Table 2:** Mean accuracy of the happy- and sad-face search tasks for the two Child Physical Abuse (CPA)-risk groups

	Target-present trials						Target-absent trials					
	1		6		12		1		6		12	
	Mean	SD	Mean	SD	Mean	SD	Mean	SD	Mean	SD	Mean	SD
Happy-Face Search Task												
Low CPA risk (n = 39)	99.1	(2.6)	96.8	(5.9)	95.1	(6.3)	99.4	(2.2)	99.6	(1.9)	99.8	(1.3)
High CPA risk (*n* = 11)	99.2	(2.5)	90.9	(7.9)	95.5	(5.7)	100.0	(0.0)	100.0	(0.0)	100.0	(0.0)
Sad-Face Search Task												
Low CPA risk (*n* = 36)	98.6	(3.1)	98.8	(2.9)	99.3	(2.3)	100.0	(0.0)	100.0	(0.0)	99.8	(1.4)
High CPA risk (n = 13)	99.4	(2.3)	98.7	(3.1)	98.1	(3.7)	99.4	(2.3)	100.0	(0.0)	98.7	(3.1)

For the sad-face search task, the target-present trial type was again analyzed using an ANOVA. Here, there was neither a main effect of CPA risk (*F* < 1), a main effect of set size (*F* < 1), nor an interaction effect (*F*(2, 94) = 1.15, *p* > .32). However, for the target-absent trial type, there was a main effect of CPA risk (*F*(1, 47) = 5.64, *p* = .022, η^*2*^_*p*_ = .107). The subsequent comparisons for the simple main effect indicated that the overall accuracy of the high-CPA-risk group was significantly less than that of the low-CPA-risk group (*t*(44) = 2.37, *p* = .021, *d* = 0.42). Neither the effect of set size (*F*(2, 94) = 2.99, *p* = .055) nor the interaction effect (*F*(2, 94) = 1.45, *p* > .23) were significant.

#### RT differences (Δ RT)

**Fig. 2:**
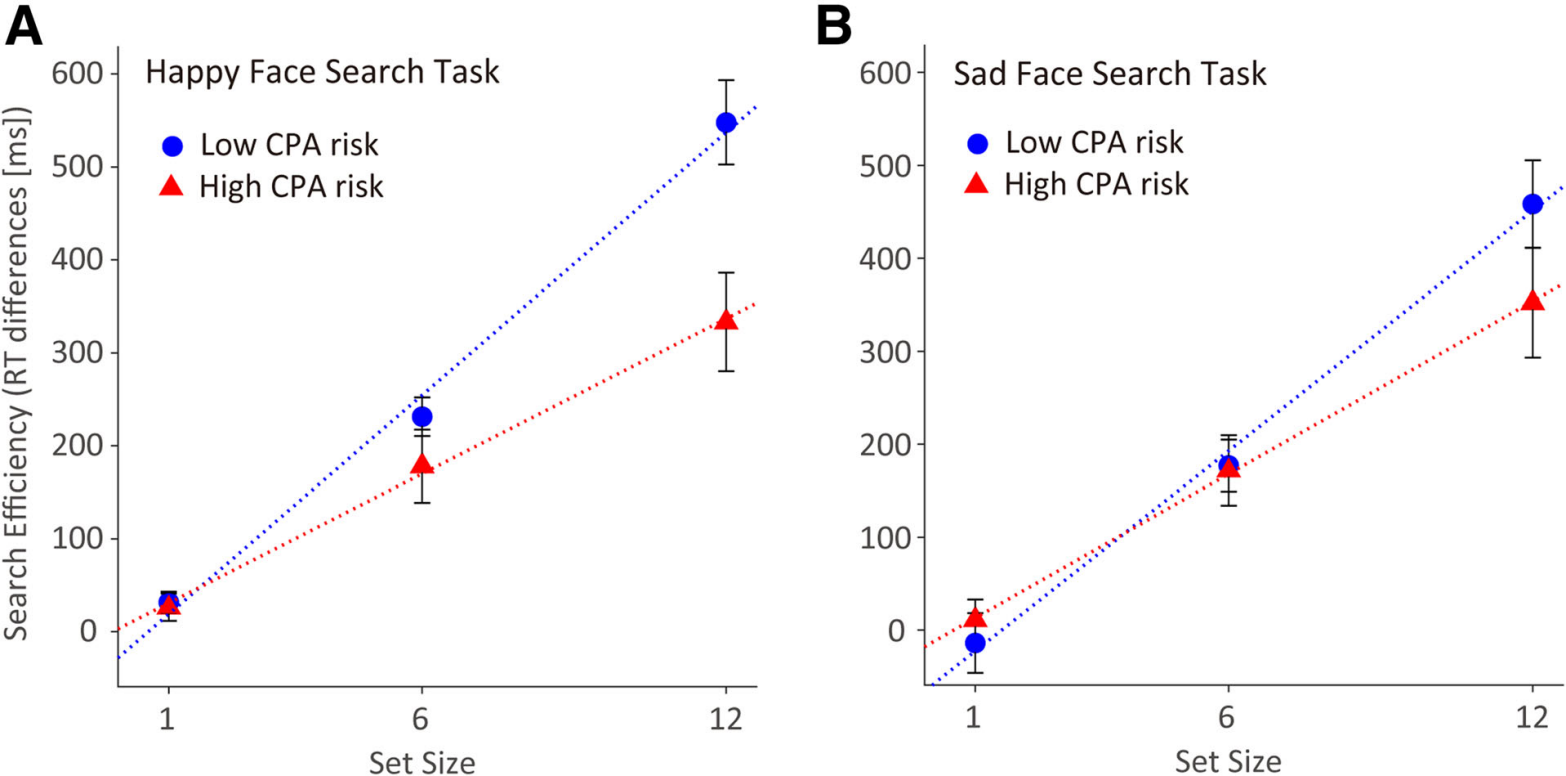
Mean differences in Reaction Time (Δ RT) and search slope (Δ search slope) between target-absent and -present trial types for the Child Physical Abuse (CPA)-risk groups. **a** For the happy-face search task, the high-CPA-risk group searched significantly less efficiently than the low-CPA-risk group. **b** For the sad-face search task, there was no inter-group difference in search efficiency. Error bars represent the standard errors of the mean

For the happy-face search task, the differences in RT (Δ RT) between the target-absent and -present trial types were analyzed using an ANOVA. Here, there were main effects of CPA risk (*F*(1, 48) = 4.44, *p* = .040, η^*2*^_*p*_ = .085) and of set size (*F*(2, 96) = 67.84, *p* < .001, η^*2*^_*p*_ = .586), as well as an interaction effect (*F*(2, 96) = 4.79, *p* = .010, η^*2*^_*p*_ = .091). As indicated by subsequent comparisons for the simple main effect, the high-CPA-risk group showed significantly less-efficiency performing the visual search for the happy face in the set of 12 than did the low-CPA-risk group (Fig. 2a; *t*(48) = 2.38, *p* = .021, *d* = 0.91). On the other hand, for the sad-face search task (Fig. 2b), an ANOVA of the Δ RT showed that there was a main effect of set size (*F*(2, 94) = 56.48, *p* < .001, η^*2*^_*p*_ = .546). Neither the effect of CPA risk (*F* < 1) nor the interaction effect (*F*(2, 94) = 1.60, *p* > .20) were significant.

#### Search slope differences (Δ search slope)

As shown in Fig. 2a and b, the differences in search slopes (Δ search slope) for the happy-face search task differed significantly between the CPA-risk groups (*t*(48) = 2.35, *p* = .023, *d* = 0.88), but not for the sad-face search task (*t*(47) = 1.44, *p* > .15, *d* = 0.49). This indicates that the high-CPA-risk group (mean Δ search slope [SD] = 27.83 [17.53]) had significantly lower search efficiency for the happy face than the low-CPA-risk group (mean Δ search slope [SD] = 47.13 [25.54]).

To further explore the relationship between the demographic and psychological characteristic data, the Δ search slopes for the happy-face search task, and the CPA-risk, we performed logistic regression analyses with the CPA-risk groups (i.e., low, high) as the binary outcomes. The Δ search slopes as well as five measures that showed significant between-group differences (i.e., number of children, the BPAQ Anger scores, the J-PSI Child domain subscores for mood and acceptability, and the J-PSI Parent domain subscores for attachment) were the predictors. The analyses showed that the Δ search slopes for happy faces (Wald = 4.63, *p* = .031, OR = 1.06, 95% CI [1.01, 1.11]) and number of children (Wald = 4.53, *p* = .033, OR = 0.22, 95% CI [0.05 to 0.89]) were significant predictors for being in the high-CPA-risk group. As confirmed by supplementary analyses using the mediational model, the two variables, Δ search slopes and the number of children, each had direct, but not indirect, effects on CPA-risk. Moreover, none of these five measures were significantly correlated with the Δ search slopes for the happy-face search task (all *ps* > .43).

#### Search strategies

To further explore the search strategies of the target-happy and -sad faces, the search slopes for the target-present trial type of the face search tasks were compared to zero in a one-sample *t*-test using a Bonferroni correction for multiple tests. For the target-happy faces, significantly increasing search slopes with more set sizes were shown in both the low- (*t*(38) = 12.14, *p* < .001) and high-CPA-risk groups (*t*(10) = 9.84, *p* < .001). On the other hand, for the target-sad faces, there were neither significant search slopes in the low- (*t*(35) = 1.07, *p* > .58) nor the high-CPA-risk groups (*t*(12) = 2.39, *p* = .068).

## Discussion

The current study examined how individual differences in CPA risk are associated with the rapid detection of positive (happy) facial expressions during a single-target face search (face-in-the-crowd) task. Based on the Δ RT and Δ search slopes between the target-absent and -present trial types for each face-search task, the high-CPA-risk group was found to be significantly less efficient at searching for a happy, but not sad, face than were the low-CPA-risk group. The self-reported face ratings of valence and arousal did not differ between the 2 CPA-risk groups. The happy and sad faces that were rated in this study were consistent with happiness and sadness on the emotional expressions distributed in the arousal-valence orthogonal dimension of the circumplex model of affect [61]. On this dimension, happiness is high in pleasantness and high in arousal, whereas sadness is low in pleasantness but low in arousal, which was what our findings showed. The current study presented evidence that higher CPA risk was associated with less efficient detection of happy facial expressions in the face-search task rather than the visual saliency of the target face among the non-target faces.

Consistent with existing SIP models regarding CPA risk, the results of the current study suggest that showing less efficient detection of positive facial expressions in the SIP system is associated with a higher CPA risk. In particular, low- and high-CPA-risk mothers differed in how they rapidly detected happy facial expressions, but not in how they explicitly evaluated them. This less-efficient detection of happy facial expressions in high-CPA-risk mothers is likely to indicate a deficiency in the initial stages of their SIP, as characterized by the four-stage model [19, 20]. In previous studies involving verbal-stimulus input [23, 24, 27, 33], such decreased processing of positive information in the SIP system were shown across several processing stages. For example, in a cued-recall task, high-CPA-risk parents recalled less positive information when cued by positive words, indicating less accessibility of positive schema during the second or third processing stage [24]. Although the current study, applying visual (non-verbal) materials, differs from previous studies in terms of its experimental paradigm, it suggests that the distorted social cognitive mechanisms underlying CPA risk are associated with early processing (detection) of visual facial expressions rather than later processing (evaluation) of the emotions depicted by the facial expressions.

Based on the results of the search strategies, the overall search slope of the target-happy, but not target-sad, faces in this study increased with more set sizes, indicating serial exhaustive search processes that were different from parallel search processes [38]. Combined with these findings, the influence of CPA risk on the efficient detection of visual facial expressions appears to vary depending on the visual search strategies (i.e., parallel or serial). According to models of visual searches [63], it is assumed that information about the presence of task-relevant features is accumulated in parallel searches (spatially global guidance) and is then used to control the allocation of spatial attention to possible target objects (spatially focal selection). A choice between parallel and serial selection strategies is assumed to be determined by the nature of a particular search task. Thus, the influence of CPA risk on the happy-face search efficiency may occur under conditions where processing demands of the task are greater; in that case, a serial selection strategy is chosen. As considered from one evolutionary perspective, mothers who could efficiently detect children’s negative signals (e.g., sad or crying expressions) as signs of distress provide a greater chance of survival for the children and, over time, a parent-child communication system developed in which children’s stylized distress signals triggered maternal attention and care [41–43]. Although detection of another’s distress generally encourages empathic (prosocial) responses, such distress signals can also often produce aversive responses, including anger, horror, and even physical abuse [64–66]; further, subclinically distressed mothers have been found to generally have lower brain function regarding their interpretation of social signals [67]. On the other hand, given that positive social signals have an important adaptive function facilitating interpersonal relationships [34, 35], less-efficient detection of happy facial expressions may decrease the likelihood of a mother having positive interpersonal experiences, consequently leading to a relatively increased probability of detecting children’s distress signals and an increased probability of experiencing frustration and stress in such situations [68, 69]. Taken together, it is possible that the serial search of happy signals may be relatively vulnerable to CPA risk, while the parallel search of sad signals may be relatively resilient to CPA risk.

Moreover, inefficient detection of happy facial expressions can also influence interpersonal experiences with other adults and children in parental caregiving contexts. Parental caregiving commonly involves social cooperation with others (i.e., co-parenting, which refers to coordination between individuals responsible for the care and upbringing of children) [70, 71]. When a person is perceived to be happy, the positivity typically spreads to the perceiver (interpersonal warmth) and, consequently, the perceiver becomes more inclined to cooperate with the person [72, 73]. In a co-parental setting, when a parent detects their partner (or social supporter) to be happy, it may cause herself/himself to selectively focus on the partner’s co-parental efforts, which may lead to improved co-parenting. Conversely, lower positive biases in the SIP system can interfere with positive co-parental experiences. For example, high-CPA-risk parents with inefficient detection of positive information may have more difficulty feeling interpersonal warmth and associating it with cooperativeness, consequently preventing themselves from fully engaging in problem-solving of family matters with their partner, which would, in turn, lead to childrearing disagreements and heightened co-parental conflicts. Children’s exposure to intense parental/co-parental conflicts has been reported to be associated with an increased risk of altered brain-development trajectories during childhood [74, 75] and into adulthood [76]. Thus, to prevent child maltreatment and related problems (e.g., co-parental conflicts), further studies are needed to identify the social cognitive mechanisms that prompt a parent to progress from positive toward negative interpersonal relationships with children and other adults in parenting/co-parenting contexts.

To date, SIP models concerning CPA risk and related study paradigms have mainly focused on explicit late-stage processes rather than implicit early-stage processes. Consequently, scientific understanding of distorted late-stage processes in at-risk parents has been applied to the design of cognitive-behavioral interventions designed to modify interpretive bias [17, 77–79]. On the other hand, the current study suggests that distorted early-stage processes in the SIP system are associated with high CPA risk. The application of this scientific evidence in parenting programs focusing on attentional bias modification (ABM) may enhance tailored interventions targeting the specific bias profiles shown by individual parents. In other research fields, it has been indicated that ABM training, which encourages positively-focused attention-search modes, reduces self-reported stress and physiological (e.g., cortisol) measures of stress reactivity [80, 81]. Such tailored interventions (e.g., ABM training) might benefit the prevention of interpersonal problems (e.g., child maltreatment), as well as providing support to families with a large number of children [82]. Although whether parenting programs for ABM effectively modify not only the attentional biases but also the parenting stress and maladaptive parenting behaviors of at-risk parents is still not fully understood, further studies of the SIP models regarding CPA risk may present avenues for the early identification and prevention of child maltreatment and related problems.

A few potential limitations of the current study should be noted. First, our high-CPA-risk group was modest in size. A post-hoc sample size calculation [83] for a two-sample *t*-test as a main analysis indicated a minimum sample size of 26 for each group (calculated effect size = .80; alpha level = .05; power = .80), and therefore, this study was slightly underpowered, thus other potentially significant findings may have been missed. Studies involving a larger number of participants are essential for generalizing our results. Second, schematic faces used here have reduced ecological validity, although many visual search studies have used schematic faces to eliminate low-level perceptual variations found in actual faces (e.g., photographs). Given this tradeoff between experimental control and ecological validity [84], future studies are needed to examine whether similar results would be obtained using photographed faces. In this study, it was important that self-reported emotional ratings of the schematic faces were fit with the emotional expressions distributed in the arousal-valence orthogonal dimension of the circumplex model of affect [61]. On this dimension, happiness is high in pleasantness and high in arousal, whereas sadness is low in pleasantness but low in arousal. Finally, the positive stimuli used in this study were only limited to happy faces (i.e., genuine smiles). As previously shown, even in the absence of happy eyes, a smiling mouth face (i.e., a nongenuine or fake smiling face) was likely to bias the judgment of the expression as being happy [85], and was associated with an increased inclination to cooperate with the smiling person [72, 73]. Further studies using an ambiguous happy-face search task with fake smiling faces would be helpful to better understanding the social cognitive mechanisms associated with CPA risk and maladaptive parenting behaviors.

## Conclusions

In this study, we found that high-CPA-risk, compared to low-CPA-risk, mothers showed less efficiency when searching for happy facial expressions; meanwhile, self-reported emotional ratings of the faces did not differ. Consistent with SIP models, our findings suggest that low- and high-CPA-risk mothers differ regarding the speed by which they detect positive facial expressions, but not in how they explicitly evaluate them. On the CPA-risk continuum, less efficient detection of positive facial expressions in the initial processes of the SIP system may begin to manifest in the mild physical discipline (punishment) stage and decrease the likelihood of producing positive interpersonal experiences, consequently leading to an increased risk of CPA and communication conflicts with others in parental caregiving settings.

## Data Availability

The datasets used and/or analyzed during the current study are available from the corresponding author on reasonable request.

## Abbreviations

ABM: Attentional bias modification
ANOVA: Analysis of variance
BDI-II: Beck Depression Inventory-II
BPAQ: Buss-Perry Aggression Questionnaire
CPA: Child physical abuse
GBVS: Graph-Based Visual Saliency
HOME: Home Observation for Measurement of Environment
ICCE: Index of Child Care Environment
IRI: Interpersonal Reactivity Index
J-PSI: Japanese version of the Parenting Stress Index
PBI: Parental Bonding Instrument
RT: Response time
SIP: Social-information processing
WBFS: Wong-Baker Faces Pain Rating Scale
